# Nanoengineered PDMS/Pd/ZnO-Based Sensor to Improve Detection of H_2_ Dissolved Gas in Oil at Room Temperature

**DOI:** 10.1021/acssensors.4c02896

**Published:** 2025-04-04

**Authors:** Glauco Meireles Mascarenhas Morandi Lustosa, Agnes Nascimento Simões, Eugênio de Souza Morita, André Nunes de Souza, Floriano Torres Neto, Waldir Antonio Bizzo, Talita Mazon

**Affiliations:** † Ministério da Ciência, Tecnologia e Inovação (MCTI) − Centro de Tecnologia da Informação Renato Archer, Campinas/SP 13069-901, Brazil; ‡ Universidade Estadual de Campinas (UNICAMP) − Faculdade de Engenharia Mecânica, Campinas/SP 13083-860, Brazil; § Universidade Estadual Paulista (UNESP) − Departamento de Engenharia Elétrica, Bauru/SP 17033-360, Brazil; ∥ HOG, CPFL Geração, Campinas/SP 13088-900, Brazil

**Keywords:** zinc oxide, hydrogen gas, nanostructured sensor, polymeric porous layer, in situ measurements, real-time monitoring

## Abstract

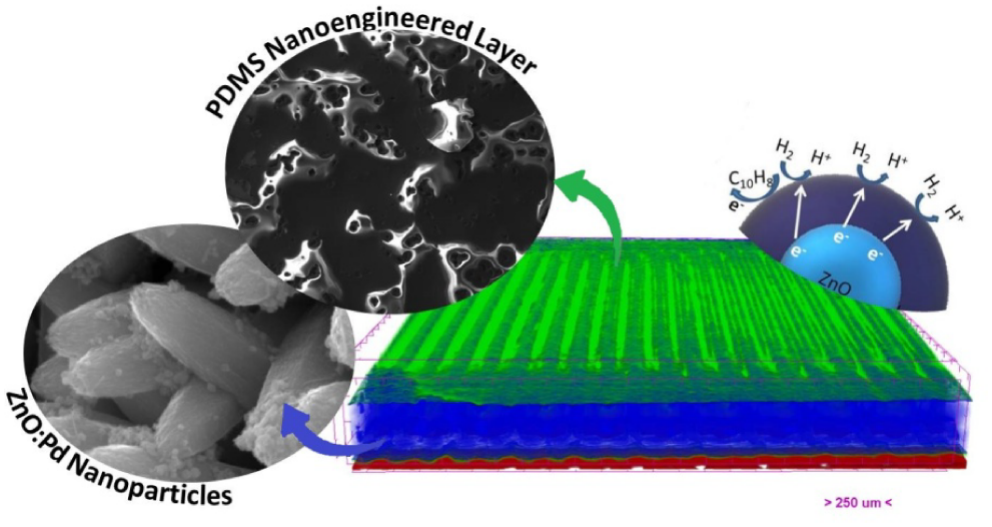

The current research aims to synthesize zinc oxide decorated with palladium nanoparticles and develop a stable sensor with high sensitivity to hydrogen gas dissolved in oil. ZnO nanorods (NR) were synthesized by a hydrothermal method directly onto a commercial sensor board with gold interdigital electrodes, followed by functionalization with Pd nanoparticles (NP) by drop casting. SEM images show ZnO NRs with an average diameter of ∼220 nm and Pd spherical NPs with diameters of 35–75 nm. Finally, the sensing properties were examined by immersing the sensor into insulating mineral oil in a closed system, where different H_2_ concentrations (from 0 up to 500 ppm) were injected into the headspace and then dissolved in the mineral oil, according to the Ostwald coefficient. All measurements were carried out at room temperature. The electrical characterization showed that our sensor had good repeatability, stability, and sensitivity to detect lower concentrations (less than 10 ppm). Additionally, a nanoengineered porous layer of PDMS was prepared over the sensor board through spin coating and heat treatment, and then the sensitivity of our sensor board reached ∼2.8 ppm of H_2_ gas. Our findings indicate that the methodology applied improves gas detection performance in industrial applications and its potential use for real-time monitoring.

The operation of transformers in the electric power industry is critical to the security and stability of power systems. Typically, power transformers use mineral oil as insulation, which can generate and release various gases during operation.^1−3^ Mainly, carbon monoxide (CO), carbon dioxide (CO_2_), acetylene (C_2_H_2_), ethylene (C_2_H_4_), methane (CH_4_), ethane (C_2_H_6_), and hydrogen (H_2_) are the characteristic fault gases that are produced and dissolved in transformer oil.^4−6^ Negative factors during the operation of power transformers, such as high temperature, high voltage, and the presence of oxygen and/or water, can lead to the production of these gases, resulting in overheating, discharge, partial discharge, and arc discharge faults.^2,7^

Hydrogen gas is produced in transformers as a result of thermal and electrical stresses on the transformer system, which can lead to the breakdown of the insulation. The presence of hydrogen gas in the transformer indicates that a fault or failure is occurring and that the transformer is at risk of catastrophic failure. Detecting the presence of hydrogen gas in a transformer is therefore critical for providing an early warning of potential faults or failures. Many analytical techniques can be used for the detection of hazardous gases, such as spectrophotometry, gas chromatography (GC), high-performance liquid chromatography (HPLC), mass spectrometry (MS), and others.^8^ This allows for preventative maintenance to be performed on the transformer, which can help to avoid more significant damage or even complete failure. Nowadays, one of the main techniques used for dissolved gas analysis is GC, which must be carried out in a specialized laboratory every six months. It is important to note that this analysis can be time-consuming and susceptible to errors (such as contamination or loss of gas, since an aliquot of the oil must be removed from its operating environment), besides being an expensive technique that requires efficient waste management for disposal.^9−11^

As H_2_ is a reducing gas, many sensors reported in the literature are based on n-type semiconducting metal oxide nanomaterials, such as CeO_2_, SnO_2_, TiO_2_, WO_3_, ZnO, and others,^12−19^ which are widely used due to their structural simplicity, low cost of production, sensitivity, good stability, and environmental friendliness.^20−22^ However, most research applies these sensor devices to the headspace of chambers. In this configuration, the process of separating oil and dissolved gas (based on the Ostwald coefficient) into the atmosphere is necessary, which delays gas detection and influences the efficiency of fault diagnosis.

Given the limitations of existing methods and the recent structural changes that demand rigorous quality and safety indices, there is a need for a new approach to the development of in situ analysis of dissolved gas in mineral oil from high-voltage transformers. These new approaches for real-time detection contribute significantly to a safe industrial environment and stable equipment operation. The development of an oil-immersed device (OID) serves as support for the early detection of dissolved gas to prevent faults that may appear in different voltage-class systems and also can help extend the life of the equipment while reducing the risk of costly downtime and repairs.^23^

Among the various n-type semiconducting metal oxides, zinc oxide nanostructured-based material possesses exciting functional properties and is considered to be the most promising resistance-based real-time gas sensor. ZnO is a direct wide bandgap semiconductor at room temperature (∼3.3 eV) and a large excitation binding energy (∼60 meV), making it a desirable material for developing various semiconductor metal oxide-based materials for many applications, such as gas sensors, biosensors, supercapacitors, photocatalysis, solar cells, and varistors.^24−31^ A limitation of using n-type semiconducting metal oxide nanomaterials as hydrogen gas sensors dissolved in transformer oil is the need for high temperatures (over 200 °C) to obtain better performance from the gas sensors. To overcome this, research is now focused on synthesizing ZnO nanostructures, such as nanoflowers, nanorods, nanoplates, and others, as their low dimensions increase surface reactivity and the number of active sites, consequently increasing the material’s electrical and sensor properties. Furthermore, it is crucial to seek improvements in cost efficiency, sensitivity, selectivity, and the development of devices for practical applications.^32−38^

ZnO nanomaterials have been widely used in gas sensing applications due to their high sensitivity and selectivity toward various gases, such as carbon monoxide, nitrogen dioxide, and hydrogen. The gas sensing mechanism of ZnO is based on its ability to change electrical conductivity upon exposure to gas. When ZnO is exposed to a gas, the gas molecules are adsorbed onto its surface and create charge carriers, which can be electrons or holes (depending on the type of gas and the specific conditions of the sensor). In general, reducing gases, such as carbon monoxide, tend to donate electrons to ZnO, creating an excess of electrons and resulting in an increase in conductivity, while oxidizing gases, such as nitrogen dioxide, tend to accept electrons from ZnO, creating an excess of holes and resulting in a decrease in conductivity.^39−42^

While zinc oxide has demonstrated promising behavior for the development of gas sensor devices, there has been an increasing trend toward studying metal doping and ceramic-polymer composites to obtain new gas sensors with the advancement of technology. By performing decoration of noble metal elements onto the ZnO surface, such as platinum (Pt) and palladium (Pd), support-metal interfacial sites are created, which can modify their electronic structure and surface properties. This could increase the concentration of oxygen vacancies and maximize the catalytic effect, improving the sensor’s response to hydrogen and lowering the detection limit.^43−46^ The interactions between the gas molecules and adsorbed species on the surface influence the width of the space charge region (depletion layer) and aid the chemisorption of gas molecules, leading to an improvement in performance. Moreover, Pd is highly chemically reactive to H_2_ even at room temperature and can reduce the activation energy for the dissociation of H_2_ molecules and store H atoms at interstitial sites.^47−50^

The preparation of nanostructured composites with the addition of polymers has been studied as an effective approach to enhance the gas-sensing properties of zinc oxide-based gas sensors, providing additional selectivity to the semiconductor-based sensor, as they can selectively interact with specific gas molecules. Moreover, polymers can improve the sensitivity of the sensors by creating a synergistic effect between the ZnO and the polymer, acting as a bridging agent between ZnO nanoparticles, facilitating the transport of electrons between them, and resulting in an increase in the sensitivity of the sensor.^51,52^

The use of polymeric composites with metal oxides brings benefits to the properties not only for the detection of dissolved gases but also in sensing performance in general. The heterostructured composite system of In_2_O_3_ nanosheets/Polypyrrole (PPy) nanoparticles^53^ was developed as an ecofriendly, self-powered sensor for the detection of nitrogen dioxide (NO_2_) with fast response/recovery time, a wide detection range, high repeatability, and long-term stability. A polyaniline/ZnO heterostructure-based^54^ gas sensor with a large specific surface area and highly active sites was prepared for NH_3_ detection, showing a minimum detection limit of 0.1 ppm, and the response rate reached 96.1% at a concentration of 10 ppm. In addition, this polymeric approach can be applied to obtain other flexible electrodes, for example, to develop sensors for human-motion monitoring with excellent tactile sensing performance, instantaneous response capability, and potential applications in self-powered systems.^55−57^

In this work, we aimed at developing a feasible and moderate fabrication route for highly sensitive H_2_ gas sensors at room temperature by constructing a nanostructured sensor based on ZnO nanorods decorated with palladium nanoparticles anchored on their surface. Electrical measurements were performed with the sensor immersed in mineral oil for the detection of H_2_ gas through variations of the electrical resistance of ZnO, analyzed continuously. To improve the performance of the sensor, a porous polymeric layer of polydimethylsiloxane (PDMS) was deposited via spin coating. This porous layer is capable of protecting ZnO particles from mineral oil and acts as a filter, enabling the detection of lower concentrations of hydrogen in the oil at ambient room temperature. Moreover, this procedure is low-cost and easy to manufacture, which makes the manufacturing process simpler and scalable.

## Results and Discussion

### Structural and Morphological Characterizations

**Figure 1:**
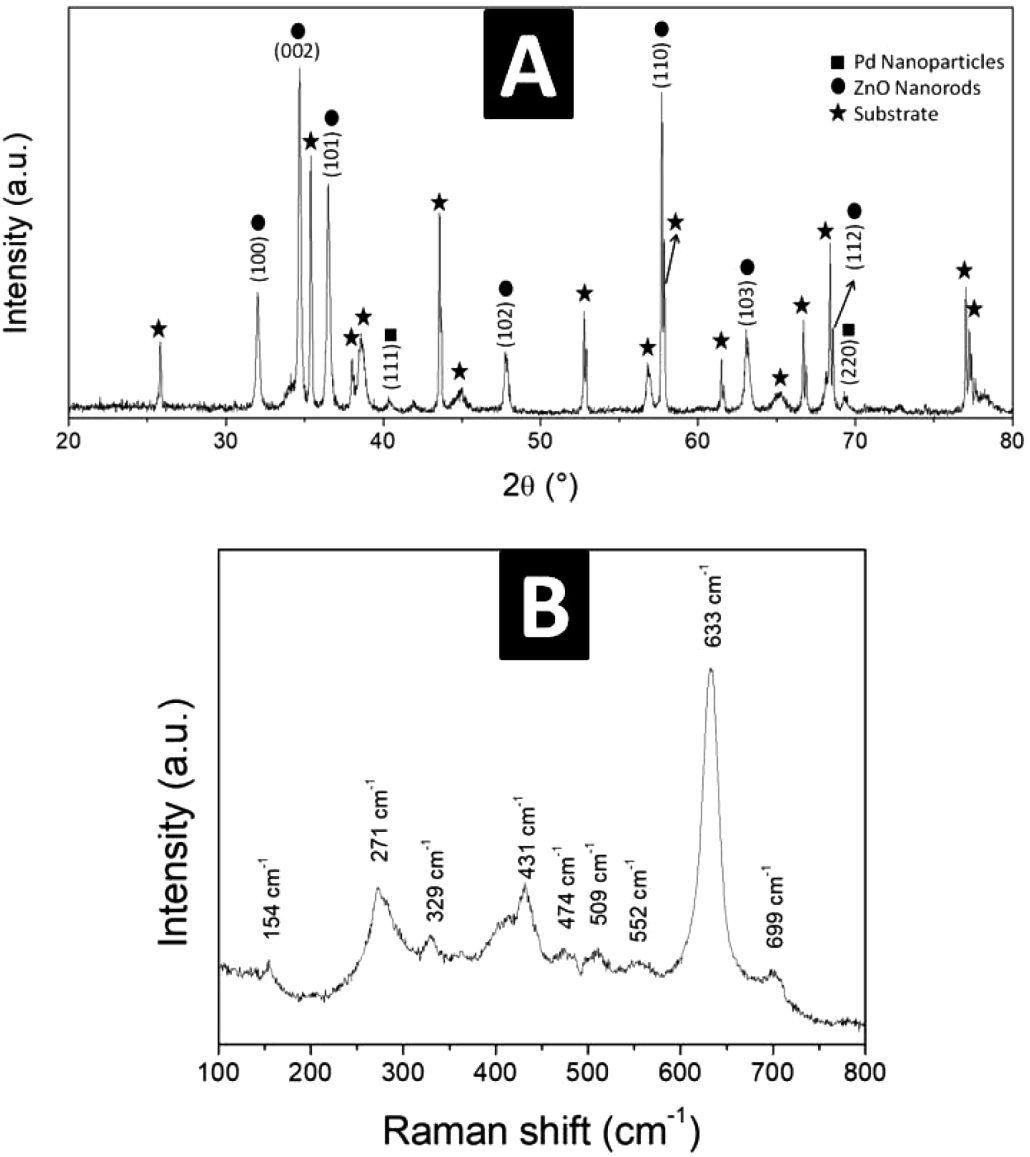
(A) XRD diffractogram and (B) Raman spectra of ZnO NR:Pd NP on the sensor board.

Figure 1A shows the XRD pattern obtained for our as-prepared base sensor. We can identify in the diffractograms crystalline peaks related to the cubic structure of Pd (111) orientation, according to JCPDS-ICDD card n° 46–1043^58^ and peaks indexed to the ZnO hexagonal wurtzite crystalline structure, according to JCPDS-ICDD card no. 36–1451. The preferential growth along the (002) plane is characteristic of ZnO nanorods with polar faces [(002) plane] and nonpolar faces [(100) and (101) planes]. The growth along the (002) plane indicates a faster growth rate than that along other directions. The high-intensity peaks (represented with a star symbol) are attributed to the alumina substrate profile.^59^

Typical Raman spectra of ZnO nanorods grown on an IDE sensor board and decorated with Pd nanoparticles are shown in Figure 1B. The peak labeled as the E_2_ vibration mode at 431 cm^–1^ was observed and is known as a Raman-active optical phonon mode, which is characteristic of the wurtzite hexagonal phase of ZnO. The peak at 329 cm^–1^ corresponds to the second-order Raman spectrum arising from zone boundary phonons (E_2_-High – E_2_-Low). The broad peaks at 271 and 509 cm^–1^ do not correspond to ZnO normal modes, which are associated with and activated by induced defects. The high-intensity peak at 633 cm^–1^ is exhibited by Pd nanoparticles, which are a Raman-active vibrational mode and a characteristic feature of PdO.^60−63^

**Figure 2:**
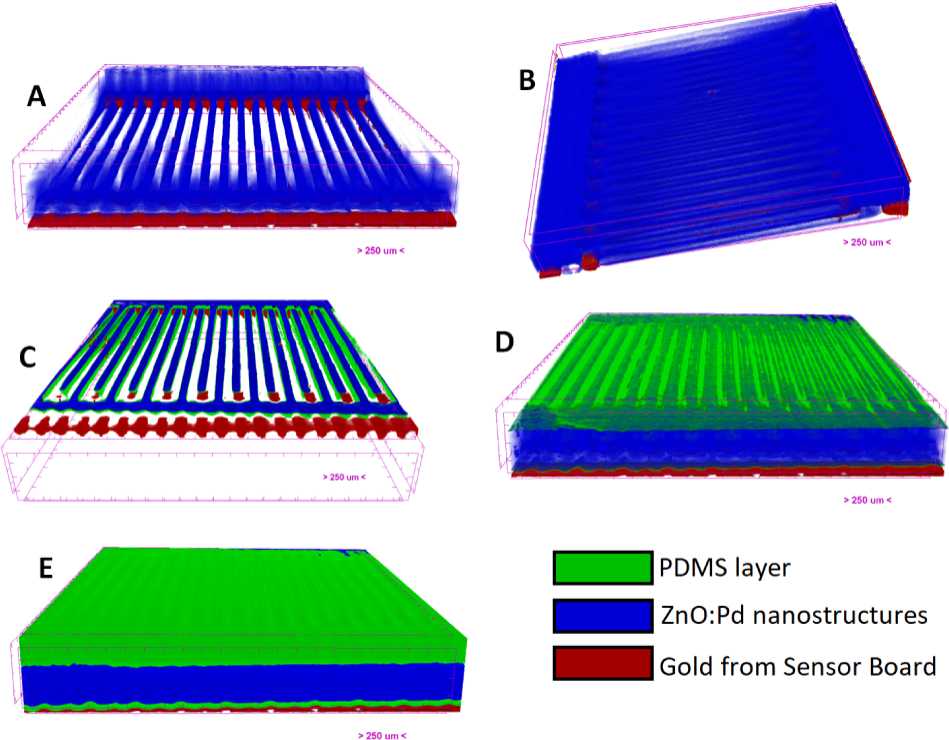
3D structural view of the ZnO NR:Pd NP:PDMS nanoengineered sensor board. (A, B) Internal view for visualization of the ZnO grown. (C) Cross-sectional view of the interdigital electrodes. (D, E) Complete view with the polymeric layer on the sensor board top, with different color intensities for viewing internal details.

The μ-CT analysis, a nondestructive 3D imaging technique, is a powerful tool for analyzing the internal structure of porous electrodes, as it can provide information on the pore size distribution, porosity, and connectivity of the electrode. A beam of X-rays passes through the sample and measures the attenuation of the beam at different angles. The attenuation is related to the density of the material; therefore, by reconstructing the attenuation data from all angles, a 3D image of the sample can be created, and the different materials can be identified. Figure 2 shows different tomography slices of the sensor board, a model in the *XYZ* plane, revealing that the zinc oxide is evenly distributed over the gold trail of the electrode (Figure 2A,B) and also reveals the PDMS nanoengineered layer (Figure 2D,E) deposited on the surface. The cross-section (Figure 2C), carried out by the equipment’s software, allowed us to observe that the PDMS layer was also deposited in the lateral area where the ZnO grew on the tracks. It was not possible to differentiate the Pd nanoparticles due to their size and quantity being below the equipment’s detection limit.

**Figure 3:**
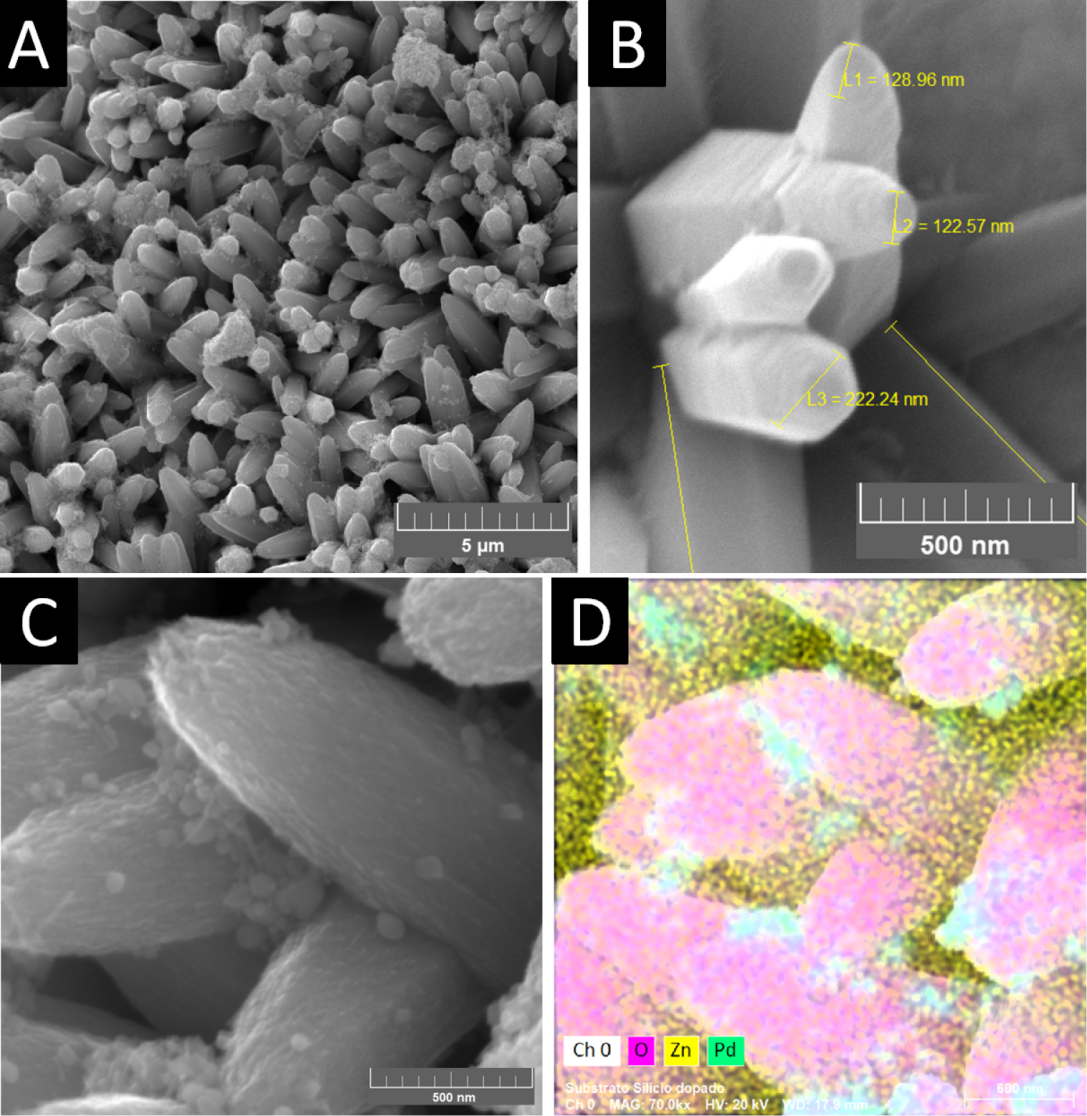
(A–C) SEM images for ZnO NR:Pd NP on the sensor board at different magnifications and (D) elemental mapping analysis from (C).

The SEM analysis was carried out in equipment of high resolution to observe the morphology of the nanostructures. Through Figure 3A,B, and analysis by ImageJ software, it can be seen that the ZnO had uniaxial growth, forming tnanorods with an average diameter of ∼220 nm in the central part of the nanostructure, while a diameter of ∼120 nm was observed at the top (indicating an interruption in the nanorod growth process). The palladium NPs, deposited on the ZnO surface, were observed with a spherical shape (Figure 3C), as confirmed by a qualitative analysis performed by EDS (Figure 3D), and their diameter was calculated in a range of ∼35–75 nm, determined through analysis by ImageJ software.

**Figure 4:**
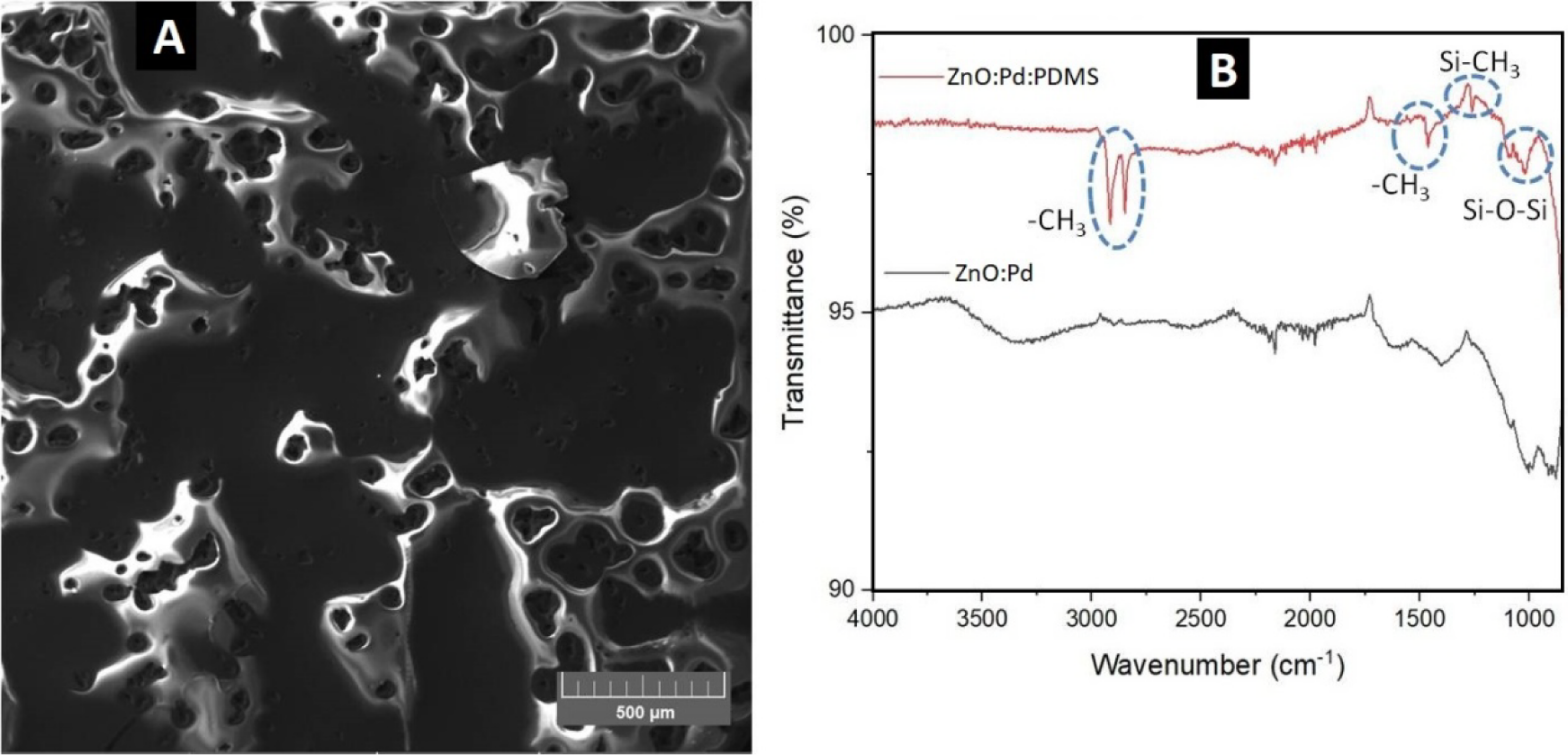
Characterizations by (A) SEM and (B) FTIR for the PDMS nanoengineered polymeric porous layer.

To verify the structural network of the as-prepared PDMS nanoengineered layer over ZnO NR:Pd NP, the sensor board was characterized by SEM (Figure 4A) and FTIR (Figure 4B). Through morphological characterization, the nanoengineered porous layer of PDSM, with a diameter calculated in the range of 60–150 μm, was analyzed using the ImageJ software. Figure 4B depicts the FTIR spectrum of the ZnO-based sensor board, which was acquired in the range of 400–4000 cm^–1^. Peaks for ZnO lattice vibrations were identified, and also for CH_3_[Si(CH_3_)_2_O]*_n_*Si(CH_3_)_3_ lattice vibrations, the PDMS formula. For the ZnO:Pd sensor board, peaks related to hydroxyl groups on the ZnO surfaces and adsorbed water molecules were identified. The band observed at ∼3450 cm^–1^ is attributed to O–H stretching vibration, and the band at ∼1650 cm^–1^ is attributed to H–O–H bending vibration. The peaks below 1000 cm^–1^ are ascribed to the Zn–O stretching bond, showing ZnO lattice vibrations and their formation, which are dependent on the type of synthesis techniques. The peaks positioned in the range between 700 and 1700 cm^–1^ are assigned to the symmetrical and asymmetrical stretching vibrations of the C—H, C—O, and C=O bonds. Additionally, some peaks related to the PDMS structure were observed: at around 1050 cm^–1^ (stretching vibration modes of the Si–O–Si bond), 1257 cm^–1^ (bending vibration of the Si–CH_3_ bond), around 2960 cm^–1^ (stretching vibration of the −CH_3_ bond), and at 1412 cm^–1^ (asymmetric deformation vibration peak of −CH_3_).^64−66^

### Gas-Sensing Mechanism and Properties

It is noticed that metal oxide (MO) semiconductor-based materials have the ability to detect gases by changing their electrical resistance when in contact with a specific gas. The gas detection mechanism is based on the interaction between the material’s electrons and the atoms of the adsorbed gas on its surface, promoting a change in its electrical conductivity, thus leading to an increase or decrease depending on the atmosphere in which the sensor is submitted, the type of gas, and its concentration. This occurs due to electron transfer between the adsorbed gas and ZnO, resulting in the formation of charge layers at the interface between the material and the gas.^67−69^

Most of the MO-based gas sensor papers available in the literature are about the detection of gases in an air atmosphere. There are few papers that focus on the real-time analysis of gases that could be dissolved into a liquid medium, such as transformer oil, due to some difficulties and limitations from transformer operations (i.e., oil flammability, risk of explosions, and risk of leaks that can poison soil and waterways).^70,71^ We must take into account that in this kind of analysis, where the sensor is immersed in mineral oil, there will be interactions between the chemical components of the oil and the sensor surface, thus promoting the interaction of electrons and the change of resistance of the device.

The oil used in our experiments has a naphthenic mineral base, i.e., it is constituted of aromatic hydrocarbons with the molecular formula C_10_H_8_. For this molecule, the electronic density is primarily concentrated in the pi-electron cloud that extends above and below the plane of the molecule. The pi-electron cloud is formed by the delocalized pi electrons present in the double bonds of the aromatic ring, shared among the carbon atoms, resulting in high electron density in the region above and below the ring plane. In addition to the pi-electron cloud, there is also significant electron density associated with the sigma bonds that connect the carbon atoms in the ring. The sigma bonds contribute to the overall stability of the molecule by holding the carbon atoms in the ring in a specific arrangement.

**Figure 5:**
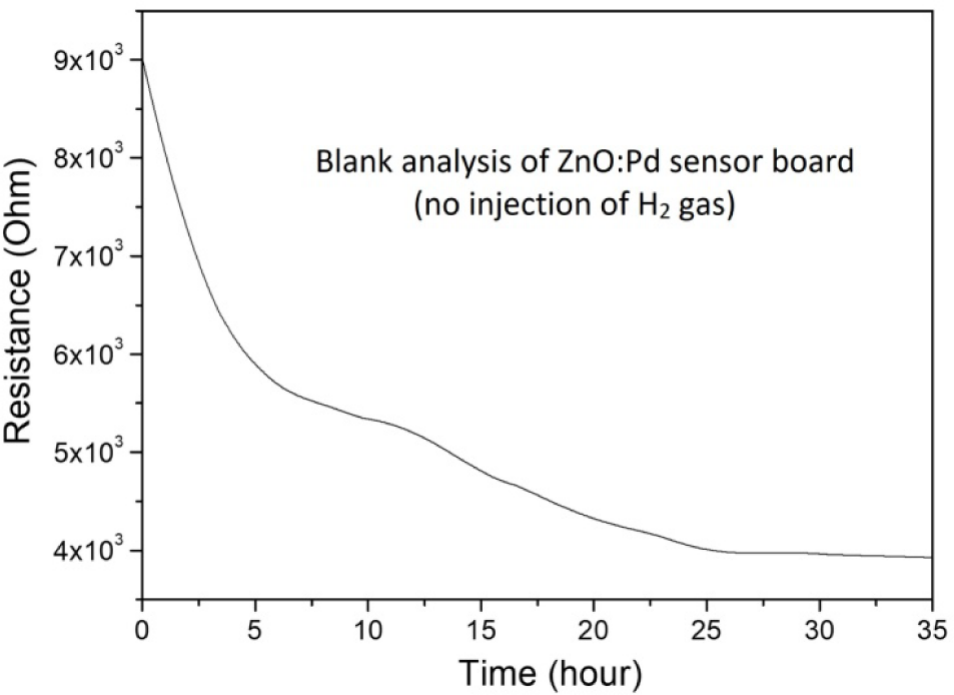
Real-time monitoring of the electrical response from the ZnO NR:Pd NP sensor board immersed in mineral oil without the injection of H_2_ gas.

Concerning the immersion of the sensor into mineral oil, the high electron density of the pi-electron cloud in the naphthalene molecules (C_10_H_8_) can interact with the ZnO surface, resulting in the transfer of electrons from the pi-electron cloud to the ZnO surface, which increases the concentration of charge carriers in the depletion layer and leads to an increase in its electrical conductivity (i.e., a decrease of electrical resistance). This behavior can be observed by performing a blank analysis. Initially, we carried out electrical characterizations over time using the sensor (without PDMS) immersed in mineral oil without any injection of H_2_ gas into the headspace chamber. The sensor response is depicted in Figure 5, demonstrating a continuous decay in its resistance as a result of interactions on the nanoparticle surface. This helps to confirm that any subsequent increase in the electrical resistance response in experiments is due to the presence of the analyzed gas, rather than being influenced by other factors.

Thereby, with the injection of hydrogen gas into the headspace of the first chamber, the environment will self-adjust to an equilibrium concentration of the dissolved gas in the liquid phase, which is achieved when the rate of gas dissolving in the liquid is equal to the rate of gas escaping from the liquid. This equilibrium concentration indicates that the concentration of a gas dissolved in a liquid is proportional to the partial pressure of the gas above the liquid. When this equilibrium concentration is reached, the oil is passed through a metallic connection, and the dissolved gas molecules are diffused into the second chamber (analysis chamber), where the ZnO-based sensor is immersed. Then, the hydrogen molecules will interact with and are adsorbed onto the surface of ZnO nanorods, withdrawing electrons from the depletion layer. This leads to a decrease in the concentration of charge carriers in the region and, thus, a decrease in electrical conductivity (i.e., an increase of resistance).

**Figure 6:**
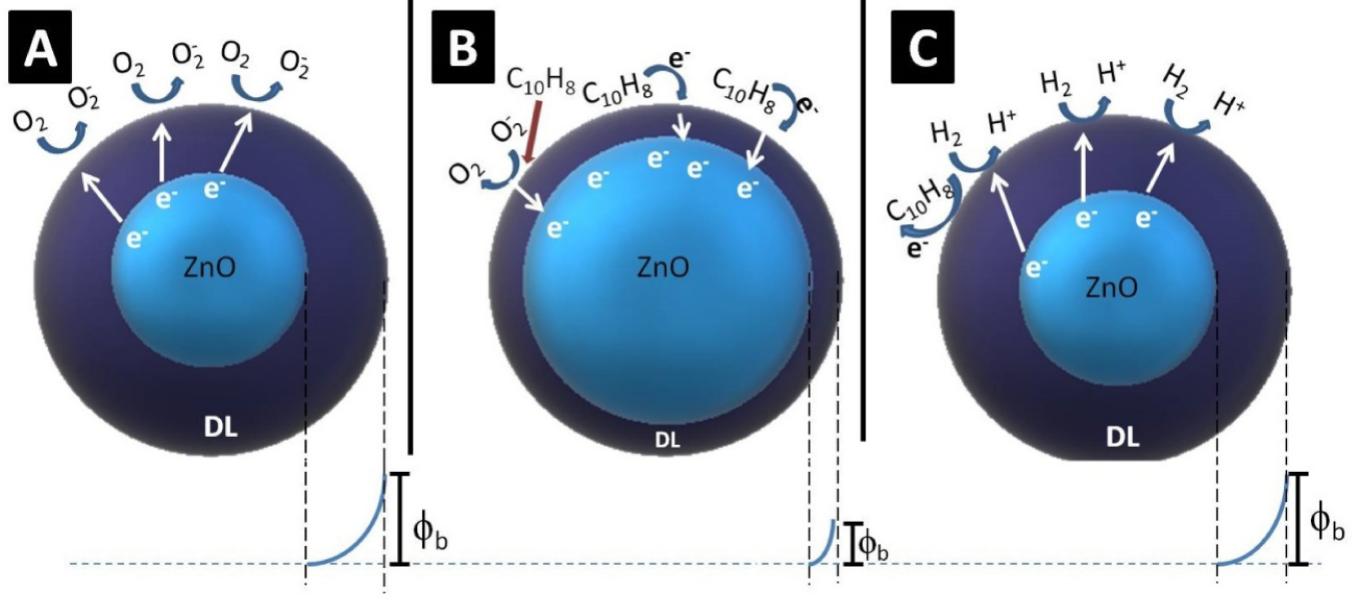
Schematic explanation for adsorption interactions on the sensor surface with modification of the depletion layer (DL) and potential barrier (ϕ_*b*_): (A) in an air atmosphere, (B) after immersion in mineral oil, and (C) with H_2_ molecules adsorption.

Figure 6 shows a simplified representation of the adsorption interactions of the molecules and their consequent modification of the depletion layer, which leads to a change in its conductivity/resistance. The depletion layer (DL) is a region in a material where there are no mobile charge carriers (i.e., free electrons or holes). This region of low carrier density is caused by the electrical potential difference at the interface between the two regions, which generates a potential barrier (ϕ_*b*_), named the Schottky barrier, which prevents carrier diffusion (electron mobility) across the interface, thus directly influencing the resistance (*R*) of the sensor material, i.e., ↑ϕ*_b_* → ↑*R*.^72−74^

The Pd NPs are well known for their high affinity for gases, such as hydrogen, and can be used as catalysts for splitting H_2_ molecules into two H atoms.^75,76^ Pd typically acts in electron sensitization because it is easily oxidized and reduced. The kinetic competition between the reduction of PdO and the reoxidation of metallic Pd (i.e., the transition of the Pd^2+^/Pd^0^ redox couple) modifies the electron arrangement on the ZnO surface, leading to changes in resistance and the corresponding gas-sensing performance. The Pd NPs can also modify the electronic band structure of ZnO (Fermi levels), creating defects and midgap states that can act as charge carriers and facilitate the transfer of electrons between H_2_ and ZnO, an important factor for determining the gas sensitivity of that material. When Pd metallic nanoparticles are exposed to H_2_ the dissociation occurs into H^+^. With the spillover effect, H^+^ can migrate from the surface of the Pd NPs to the metal oxide support, promoting the chemical reaction process. The H atoms react on the ZnO surface, trapping electrons and the conductivity decreases owing to the increased gap of depletion layer within the ZnO.^47−81^

**Figure 7:**
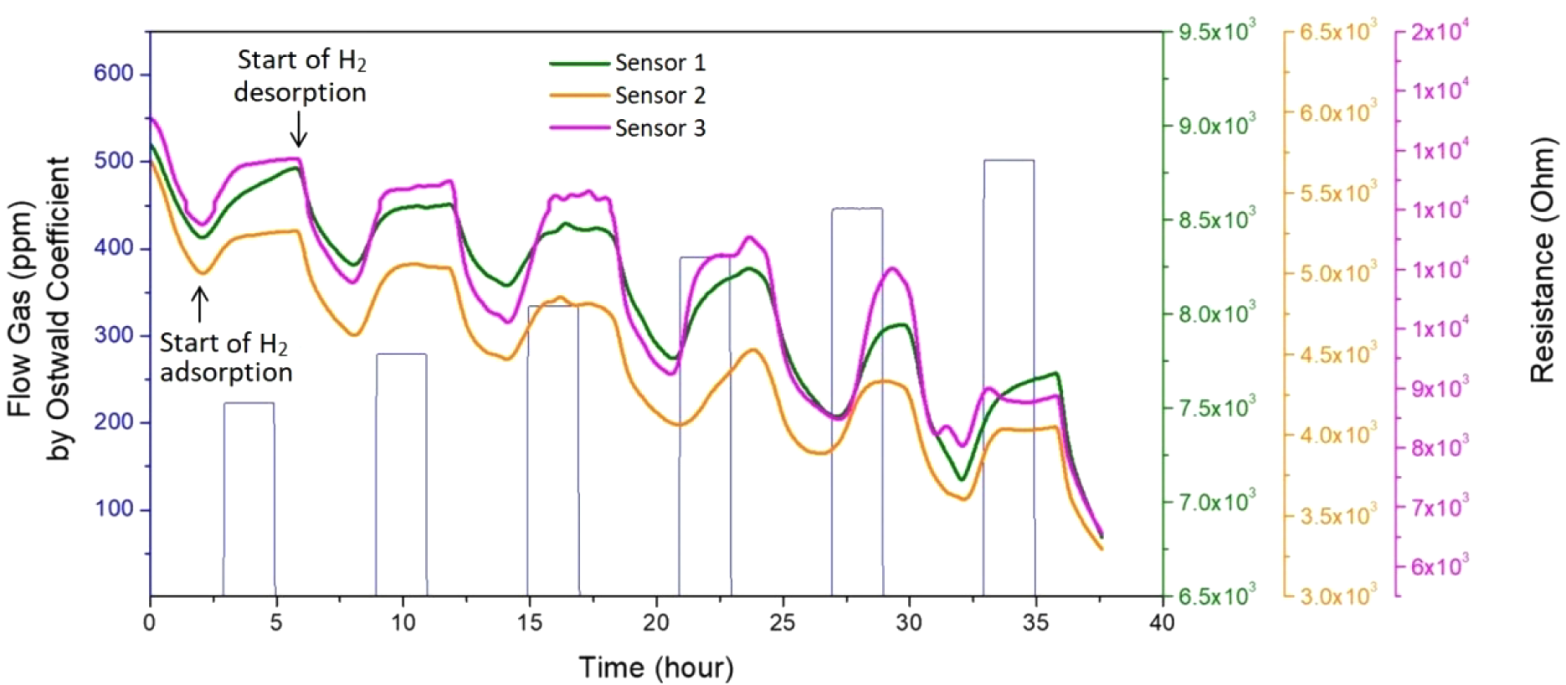
Electrical behavior toward immersion of the ZnO NR-Pd NP sensor board in mineral oil with different concentrations of H_2_ at 25 °C.

Figure 7 shows the electrical characterizations for the ZnO NR:Pd NP-based sensors prepared in triplicate (named Sensor 1, Sensor 2, and Sensor 3). In these experiments, concentration parameters in a range of 4000–9000 ppm were injected into the headspace, which corresponds to a real concentration of 223.2 ppm, 279 ppm, 334.8 ppm, 390.6 ppm, 446.4 ppm, and 502.2 ppm of H_2_ dissolved into mineral oil, after correction by the Ostwald coefficient . As observed, the baseline shows a constant decrease over time. However, at the beginning of the adsorption of hydrogen gas molecules onto the sensor surface, the resistance of the sensor increases due to changes in the depletion layer, as indicated in Figure 6. This increase in resistance can be observed as a peak in the electrical response of the sensor. It is important to note that since our experiments are performed with the sensor immersed in the oil, the adsorption–desorption processes occur more slowly due to the need for dissolution and homogenization of these gases in the analysis medium. It is also worth highlighting that as the sensor board is immersed in an environment rich in other chemical molecules that are in constant adsorption on the ZnO surface (leading to a change in electrical behavior, as shown in the blank analysis in Figure 5), it results in a sensor graph with characteristics of a p-type semiconductor.

**Figure 8:**
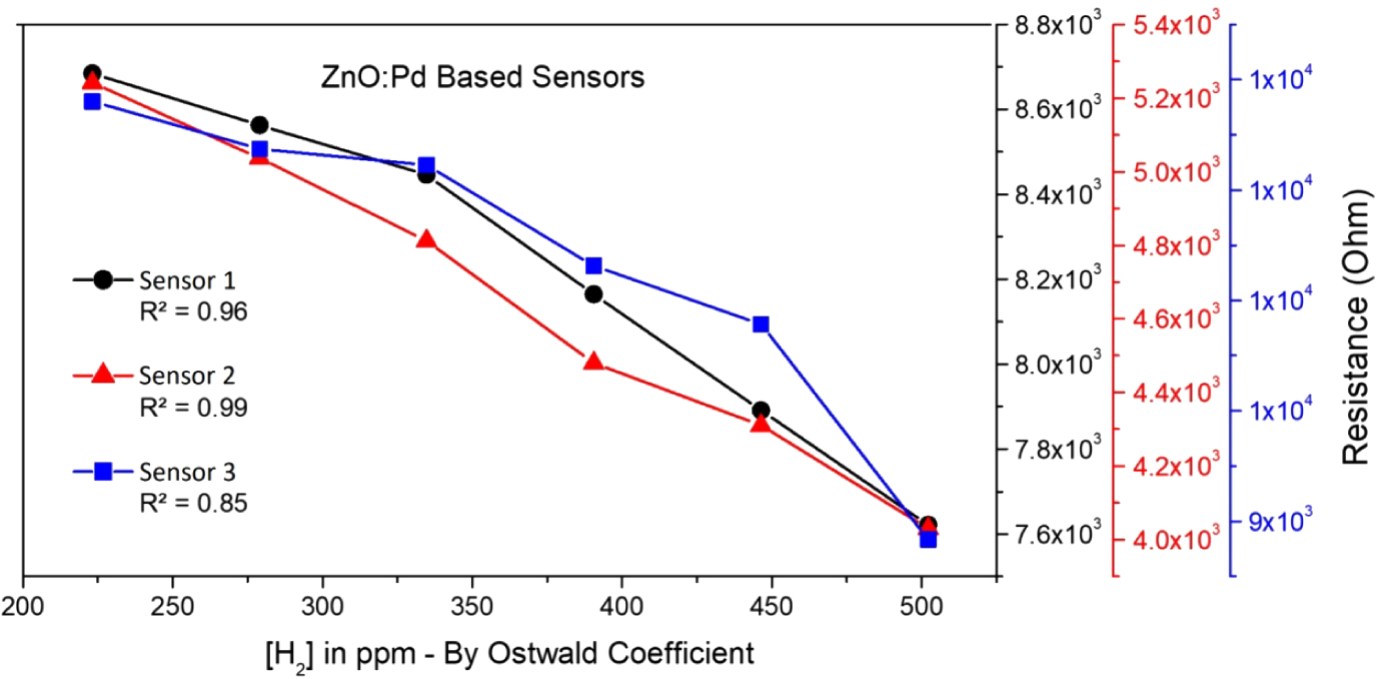
Average resistance values determined from Figure 7 for the ZnO NR-Pd NP sensor board immersed in mineral oil with different concentrations of H_2_ at 25 °C.

From Figure 7, the resistance values were calculated as the average value from the peaks of each curve. Based on this determination, a graph was plotted in Figure 8, which clearly shows a quasi-linear decreasing trend. Further, linear regression analysis was used to calculate the fit linear curve and shows a very good linear response for Sensors 1 and 2 upon exposure to various concentrations of the samples prepared in triplicate.

Other parameters that can be obtained from the graphs of Figure 7 are the Response and Recovery Time (*R*_p_ and *R*_c_, respectively). The *R*_p_ time of a sensor is defined as the time taken by the sensor to reach from 10% to 90% of the output signal, and vice versa for *R*_c_ time.^82^ The measured values of *R*_p_ and *R*_c_ times for the ZnO NR:Pd NP-based sensor upon exposure to different H_2_ concentrations are shown in Table 1. For the analyzed samples, it was observed that the *R*_p_ time (H_2_ adsorption process) was smaller than the *R*_c_ time (H_2_ desorption process) across all concentration parameters. Some factors may be contributing to this, and it is worth pointing out that (1) H_2_ adsorption may have a lower activation energy than desorption (i.e., the minimum energy required for adsorption is lower than that required for desorption; thus , with lower energy barriers, adsorption tends to proceed faster); and also (2) the presence of specific functional groups on the material surface can facilitate faster adsorption by providing favorable binding sites for hydrogen molecules. However, these same sites might also lead to stronger interactions, making desorption more difficult.

**Table 1:** Response and Recovery Time (and AV–Average Values) for the ZnO Nr:Pd NP Sensor Board

		H_2_ concentration (ppm)						
Sensor	Parameter	223.2	279	334.8	390.6	446.4	502.2	A.V. (h)
Sensor 1	Response Time (h)	1.17	0.94	1.2	1.16	0.97	1.23	1.1
	Recovering Time (h)	1.6	1.36	1.53	1.54	1.55	1.27	1.45
Sensor 2	Response Time (h)	0.9	1.2	1.06	1.4	1.26	1.07	1.19
	Recovering Time (h)	1.61	1.17	1.5	1.2	1.4	0.93	1.24
Sensor 3	Response Time (h)	1.13	0.83	1.27	0.93	1.07	0.93	1.01
	Recovering Time (h)	1.58	1.16	1.56	1.3	1.13	1.23	1.31

**Figure 9:**
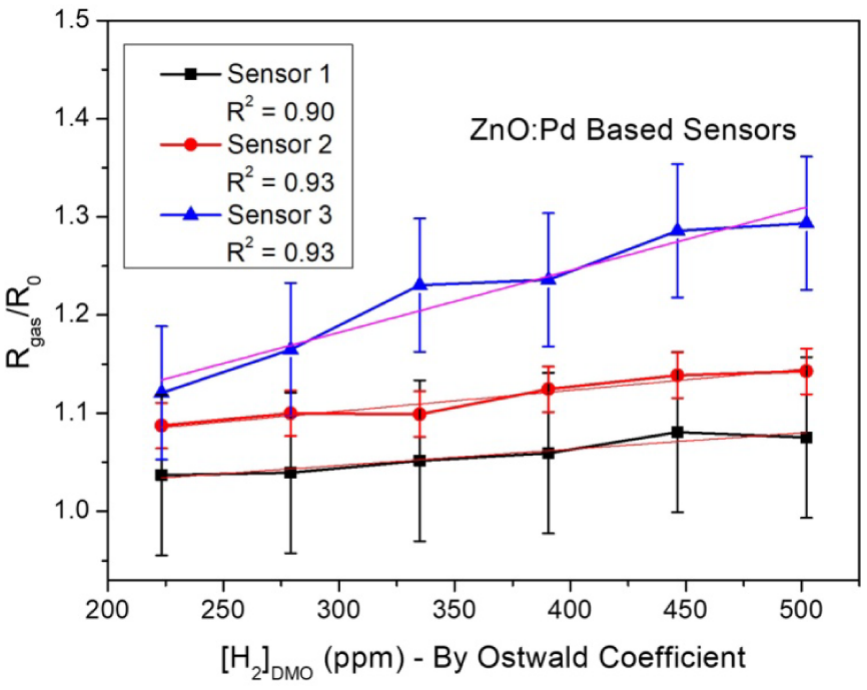
ZnO-Pd sensor response (*R*_gas_/*R*_0_) toward different concentrations of H_2_ at 25 °C.

From the data in Figure 7 it was also possible to determine the sensor response of these samples when immersed in oil-dissolved hydrogen gas. The sensor response (*S*), determined by the eq 1(83,84) and shown in Figure 9, is a relative measure of the response of a gas sensor and involves the relationship between the electrical resistance of the sensor in the presence (*R*_gas_) and in the absence (*R*_0_) of the analyzed gas. This measure can be used to compare and evaluate the sensor’s sensitivity. The curves for Zn NP:Pd NP sensor board samples showed a good fit (*R*^2^ value over 0.9). An error bar was added to the calculated points according to the standard deviation from each sample’s data, where it was determined that the sensors have low values (0.018 for Sensor 1, 0.023 for Sensor 2, and 0.068 for Sensor 3), suggesting that these sensor results are statistically significant, i.e., the data were precise and reliable.
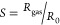
1

To improve the sensor response and be able to detect extremely low concentrations of hydrogen gas, deposition of a porous PDMS layer through *spin coating* (to promote uniform and adherent coverage on the surface of the ZnO NR:Pd NP sensor) was performed, as described in Polymeric Layer Deposition from Experimental Section. It is important to point out that the spin coating technique is relatively simple and low cost compared to other deposition methods. This facilitates the scalability and large-scale manufacturing of a porous polymer layer on ZnO NR:Pd NP sensors, making them viable for applications in diverse sectors, such as the chemical industry, environmental safety, and air quality monitoring. The use of the polymeric layer was intended as a protective membrane against naphthenic oil molecules, improving the sensitivity of the ZnO sensor and leading to more accurate and reliable detection of target gases, even at low concentrations.

**Figure 10:**
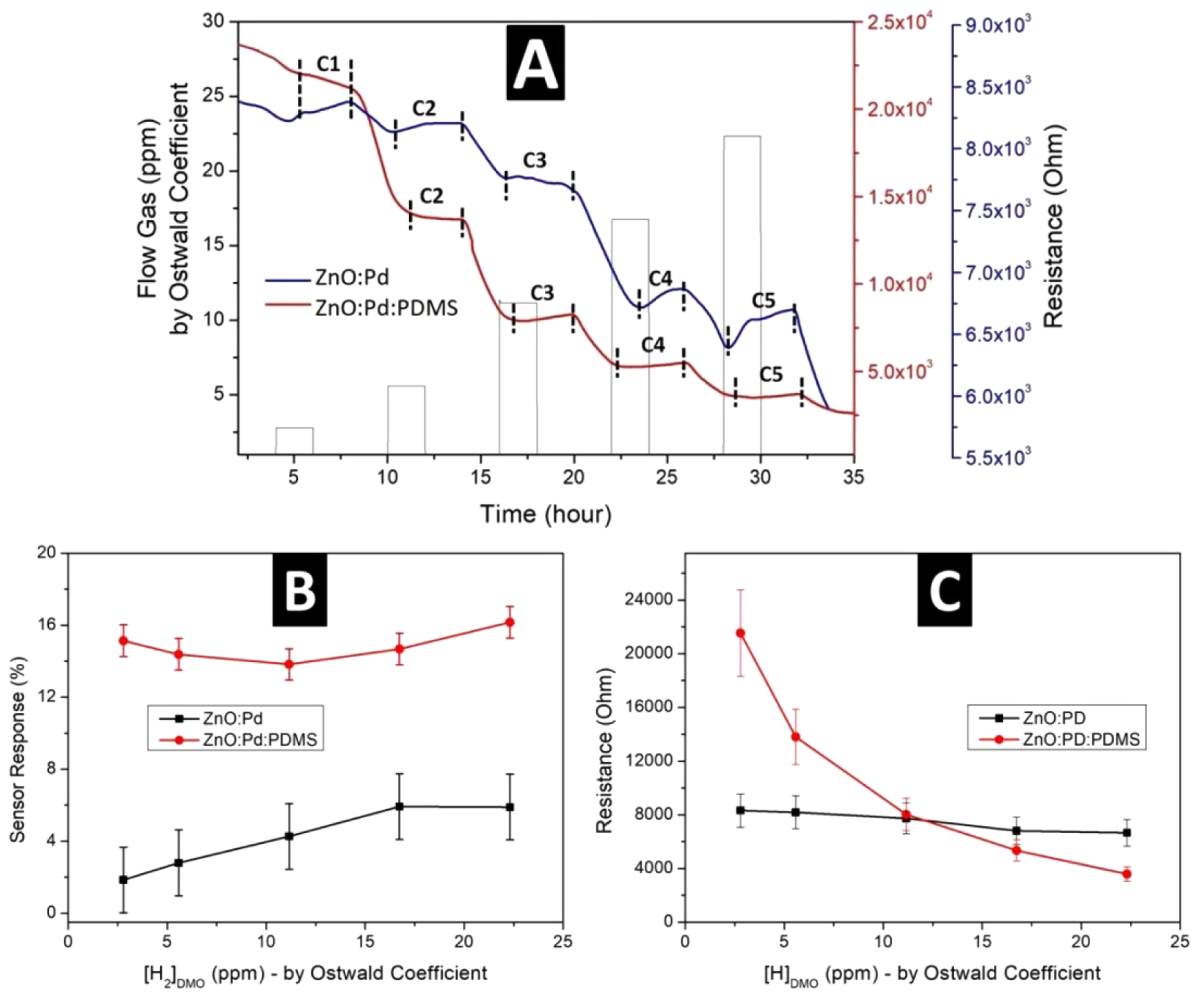
Comparative graphs of (A) electrical behavior, (B) sensor response (%), and (C) average resistance values for the ZnO NR:Pd NP and ZnO NR:Pd NP:PDMS sensor board immersed in mineral oil toward different low concentrations of H_2_ at 25 °C.

The approach used in our research showed promising results, as presented in Figure 10, in terms of signal stability and sensitivity. To compare the results with ZnO:Pd:PDMS, experiments were also carried out with the sensor board without the nanoengineered polymeric layer. Sensor 2 was chosen due to its better linear fit as well as lower standard deviation, as indicated in Figure 9. These sensors were electrically characterized with a concentration range of 50 to 400 ppm, which corresponds to 2.79 ppm (C1), 5.58 ppm (C2), 11.16 ppm (C3), 16.74 ppm (C4), and 22.32 ppm (C5). For better visualization, the graph marked the areas corresponding to each of the analyzed concentrations, due to the slight delay in the sensor response caused by the slow process of diffusion of the gas in the oil.

As observed in Figure 10A, for the resistance response of the ZnO NR:Pd NP porous PDMS layer nanoengineered sensor (ZnO:Pd:PDMS), the curve was more established and clean, with the absence of noise compared to the sample without the polymeric layer (ZnO:Pd), which favors the development of a methodology for device fabrication. For both samples, it was possible to detect ultra-low concentrations (∼2.79 ppm) of hydrogen gas dissolved in mineral oil. The sensitivity of the sensor response (%) was measured by eq 2 and plotted in Figure 10B.^82^ In Figure 10C, from the average resistance values for each concentration, it was possible to verify that the response for H_2_ detection by using the sensor board with a PDMS porous layer resulted in a significant improvement, mainly for electrical signals at concentrations below 10 ppm. While the ZnO:Pd sensor board showed resistances of 8314.98 Ω and 8185.15 Ω for C1 and C2 (i.e., 2.79 and 5.58 ppm, respectively), the ZnO:Pd:PDMS sensor board shows 21542.02 Ω and 13804.20 Ω for C1 and C2, thus promoting better differentiation between these parameters.
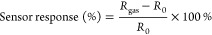
2

Through this graph, the improvement in sensor response when a nanoengineered porous PDMS layer was applied to the ZnO NR:Pd NP surface is perceptible, being better at detecting extremely low concentrations of H_2_ gas; i.e., at ∼2.8 ppm of hydrogen gas, the sensitivity is improved from almost 2% to over 15% at room temperature. As PDMS forms a film over the ZnO:Pd surface, the layer acts as a barrier, selectively allowing the target gas to reach the sensor while blocking interfering molecules or environmental contaminants, while the pores potentially increase the active surface area of the sensor exposed to hydrogen gas. This can directly lead to more interaction points that improve the signal-to-noise ratio and increase sensitivity. In addition, the functional groups (silanols) present in PDMS can interact with certain molecules, potentially enhancing their binding to the sensor surface and thus creating a more sensitive response. Statistical analysis revealed a standard deviation of 1.867 for the ZnO:Pd sensor board and 0.879 for the ZnO:Pd:PDMS sensor board. These values were incorporated as error bars in Figure 10B. The significantly lower standard deviation for the ZnO:Pd:PDMS sensor board demonstrates its enhanced accuracy and reliability compared to the ZnO:Pd sensor.

By performing bibliographic research, we found a few papers on the topic covered here in this article, where we conduct in situ detection of H_2_ dissolved in mineral oil, i.e., with the sensor immersed in the oil during analysis. In Table 2, we summarize the information on sensors for H_2_ from these papers. Many papers mainly explored promising materials using density functional theory (DFT) without conducting experimental validation for real-world applications. This is a relatively new scientific approach; thus, performing comparative studies is challenging due to the limited quantity of research using similar sensing materials and methodologies. While some sensors operate at elevated temperatures to improve their response, our sensor operates at room temperature (25 °C), significantly reducing energy consumption and simplifying device integration. As ican be observed, although we found some papers with room-temperature operation, our sensor’s limit of detection (2.8 ppm) is competitive, especially when taking into account the processing methodology, which uses somewhat complex steps and equipment to prepare the final device configuration. The use of a nanoengineered PDMS porous layer over the ZnO:Pd sensor board offers potential advantages in terms of protecting the sensing layer, which is not present in most of the compared sensors, in addition to ease of production and operating conditions. This makes it a promising candidate for H_2_ detection in real-time analysis at room temperature and for the development of immersed sensors in oil environments for industrial processes and critical systems, especially in energy industries (transmission and distribution power grid systems).

**Table 2:** Comparative Results on H_2_ Gas Sensing from Resistance-Based Sensors Reported in the Literature

Material	Processing Procedure	Analysis Conditions	Detection Limit	ref.
ZnO:Pd:PDMS	ZnO nanorods synthesized by the hidrothermal method onto interdigital electrode	Immersed sensor in mineral oil	2.8 ppm	Our Work
	Pd obtained by the solvothermal method and deposited by drop casting	Room temp. (25 °C)		
	Heat treatment at 300 °C/4 h			
	PDMS layer deposited by spin coating, followed by heat treatment at 95 °C/10 min			
ZnO:Pd	ZnO synthesized by sol–gel method	Immersed sensor in mineral oil	5 ppm	(2)
	Deposition of Pd from RF sputtering	40–80 °C		
SnO_2_–TiO_2_	SnO_2_ synthesized by the calcinations method	Sensor in atmospheric test chamber	20 ppm in 2 s	(85)
	TiO_2_ QD^a^ obtained by the solvothermal reaction	400 °C		
	TiO_2_ QDs-SnO_2_ mixed at 60 °C for 12 h			
	Deposition on IDE by screen printing			
CeO_2_–TiO_2_	CeO_2_ synthesized by chemical reaction at 150 °C/24 h	Sensor in atmospheric test chamber	10 ppm in 1 s	(86)
	TiO_2_ QD^a^ obtained by the solvothermal reaction	100 °C		
	Dissolution in ethanol and mixed at 60 °C/12 h to obtain the TiO_2_–CeO_2_			
Pd film	Pd thin film produced by vacuum evaporation and deposited on a glass plate	Pressed down to a window with the Pd film exposed to the oil	200 ppm	(87)
		20 °C		
Pd	Pd nanowire obtained through electrodeposition, followed by annealing at 200 °C/5 h in a vacuum oven	Immersed sensor in mineral oil	1 ppm	(88)
		40 °C		
SnO_2_–Pd_4_	SnO_2_ nanowires produced by electrospining	Sensor in the headspace	5 ppm	(89)
	Pd_4_ cluster obtained by magnetron sputtering	200 °C		
	Annealing at 600 °C for 3 h			
Pd–Ni (3%)	Si3N4 deposited on quartz wafer by PECVD^b^	Immersed sensor in mineral oil	10 ppm	(90)
	Ti/Pt deposited by electron beam	Room Temp. (25 °C)		
	Ti/Pd–Ni deposited by e-beam evaporation			
	Al_2_O_3_ deposited by ALD^c^			
	Selective etching of SiO_2_ and Al_2_O_3_ using RIE^d^			
Pd-coated Si	Si nanowires synthesized by top- down AEE^e^	Immersed sensor in mineral oil	1 ppm	(91)
	Pd layer deposited by ultrahigh vacuum DC sputtering	20 °C		
Pd-coated SnO_2_	SnO_2_ nanorods synthesized by e-beam evaporator	Immersed sensor in mineral oil	0.3 ppm	(92)
	Pd layer deposited by ultrahigh vacuum DC sputtering	20 °C		

a QD = quantum dots.

b PECVD = plasma-enhanced chemical vapor deposition.

c ALD = atomic layer deposition.

d RIE = reactive ion etching.

e AEE = aqueous electroless etching.

## Conclusions

In the present work, Pd-decorated ZnO nanostructures have been synthesized for the development of a sensor board of H_2_ gas. It is important to emphasize that the addition of Pd NPs to the ZnO NR sensor promotes improvements in gas detection properties compared to the unmodified ZnO sensor. Because several components in the mineral oil also adsorb onto the surface of the zinc oxide, the sensing mechanism has been continuously discussed. To improve the sensor response at room temperature, a porous PDMS layer was nanoengineered and prepared for the investigation of gas-sensing performance. The μ-CT results provide valuable insights into the internal structure of porous networks in our ZnO:Pd:PDMS-immersed gas sensor board, which can be useful to optimize the design and performance of these sensors for a variety of applications. The sensor showed good stability and sensitivity toward extremely low concentration H_2_ gas at approximately 2.8 ppm, measured at room temperature—an excellent response as a result of a synergistic effect between the catalytic and hydrogen adsorption properties of the metal–semiconductor Schottky junction between Pd and ZnO. All these findings show that the as-prepared low-cost ZnO NR-Pd NP:PDMS-porous can be promising materials for gas sensors and make a significant contribution to the industrial applications of hydrogen real-time detection.

## Experimental Section

### Chemicals

The reagents used were: zinc nitrate (Zn(NO_3_)_2_·6H_2_O, Sigma-Aldrich), ammonium hydroxide (NH_4_OH, Dinâmica), sodium tetrachloropalladate (Na_2_PdCl_4_, Sigma-Aldrich), polyvinyl pyrrolidone (PVP (C_6_H_9_NO)_*n*_, Sigma-Aldrich), diethylene glycol (Synth), and polydimethylsiloxane (PDMS, Sigma-Aldrich). All chemical reagents, commercially available, were of analytical grade.

#### Synthesis of ZnO Nanorods (NRs) on the Sensor Board

To perform hydrothermal synthesis and growth of zinc oxide on a commercial sensor board containing gold interdigital electrodes (IDE), ∼1.78 g of Zn(NO_3_)_2_·6H_2_O was dissolved in distilled water in a Teflon reactor containing the sensor base, followed by the addition of 6.5 mL of ammonium hydroxide. The sensor board used was made of an alumina substrate (10 × 30 mm size) with an IDE consisting of interspaced gold trails of 150 μm. The system was kept under stirring and heated to 90 °C for 2 h to promote the growth of nanostructures on the electrode. At the end of the synthesis, the sensor board was removed and then washed abundantly with deionized water, followed by drying at 60 °C for 15 min in a vacuum oven.

#### Synthesis of Pd NanoParticles (NPs)

The palladium-NPs were synthesized using 2 different solutions: (1) polyvinylpyrrolidone (PVP) was solubilized in diethylene glycol under magnetic stirring, then transferred to a volumetric flask coupled to a reflux system, kept under stirring, and heated at 160 °C; (2) sodium tetrachloropalladate (Na_2_PdCl_4_) was dissolved in diethylene glycol in an ultrasonic bath and then added to the reflux flask containing the PVP solution. The mixture was kept under stirring and heated at 160 °C for 1 h. Thereafter, the flask was removed and cooled in an ice bath, and the solution was centrifuged (12000 rpm for 10 min) and washed with isopropyl alcohol until complete suspension clearing and precipitation of Pd NPs.

#### Modifications of ZnO NRs Surface with Pd NPs

Pd NPs were deposited on the surface of the ZnO NRs via drop casting. An aliquot of 20 μL from the as-prepared Pd NP suspension ([Pd] = 1 mg/mL) was deposited on the sensor board, followed by drying on a heating plate at 100 °C for 5 min. This deposition procedure was performed 8 times more. Finally, the sensor board was dried overnight in a vacuum oven at 100 °C. Afterward, it was subjected to heat treatment at 300 °C for 4 h in a muffle furnace.

#### Polymeric Layer Deposition

To improve the sensitivity (i.e., detection capability) of our ZnO:Pd as-prepared device and also to reduce interference by other chemicals (from the analyzed system), we performed, in addition, a deposition of a porous polymeric layer on the ZnO NR:Pd NP surface using polydimethylsiloxane (PDMS). The bicomponent polymer was prepared in a 1:10 ratio and then mixed with finely sieved sucrose (74 μm) and toluene. The dispersion was then deposited on the ZnO NR:Pd NP surface via *spin coating* and then rotated at 7000 rpm for 30 s, followed by heat treatment at 95 °C for 10 min on a hot plate to promote the curing of the polymer. Afterward, the sensor surface was gently washed for 4 min with 1 M acetic acid to eliminate the sucrose and then with distilled water. Finally, the sensor was dried at 60 °C overnight in a vacuum oven.

### Characterizations

XRD (X-ray diffraction) was performed by an XRD7000 Shimadzu X-ray diffractometer (2θ = 10 to 80° range with an increment of Δ2θ = 0.02°, Cu *K*α radiation, λ = 1.5460 Å). The crystal structure of the samples was compared to that of JCPDS card patterns.

SEM (scanning electron microscopy) was performed on a Tescan model Mira 3 XMU to evaluate the morphological parameters (size and shape) of the powders. EDS (energy-dispersive spectroscopy), employed in conjunction with SEM, was used to provide a qualitative analysis of the elemental composition (mainly for palladium deposition verification).

Raman spectroscopy measurements were performed using a Horiba Jobin Yvon spectrometer, model T64000, within the spectral range of 100 to 4000 cm^–1^ (5 scans) and using a 532 nm laser. The surface groups were characterized through infrared analysis by a Spectrum 100 FT-IR spectrometer (PerkinElmer) in reflective mode with a 4 cm^–1^ resolution and recorded from 500 cm^–1^ to 4000 cm^–1^.

X-ray microtomography (μ-CT) was performed using a Skyscan 1272 CMOS from Bruker. Scan settings were optimized with parameters appropriate for subsequent analyses (filter: Al 1.0 mm, voltage: 70–80 kV, acquisition time: ∼1h 30 min, pixel size: 10 μm). In sequence, NRecon and CTVox software were used for reconstruction into a three-dimensional model. All images have been adjusted to exclude the outer borders and darker areas caused by X-ray interference from the sample holder, and a region of interest (ROI) was selected for processing and analysis through hand segmentation followed by color distinction.

#### Gas-Sensing Measurements

The schematic representation of the equipment used to conduct the measurement process of the sensor’s properties was described in our previous work^93^ and is shown in Figures S1 and S2. A mixture of H_2_ and N_2_ is continuously injected into the first chamber (mixing tank or control chamber) by using a mass flow controller (MFC—MKS-GE50A), directly into the oil through a bubbler (made of a sintered stainless steel filter with a 10 μm passage) to increase rapid dissolution, solubilization, and equilibrium of the gases in the mineral oil, according to the Ostwald solubility coefficient. The oil is then passed through a metal connection to a subsequent second chamber (Sensor Test Tank or analysis chamber), where the sensor is immersed in the oil. A pump promotes the recirculation of the liquid between the chambers. Continuous injection of the gas mixture ensures a constant H_2_ concentration in the headspace. Excess gas from the headspace exits through a vent, where it is possible to monitor the gas flow rate (100 mL/min).

It was used ∼8.7 L of an insulating mineral oil (LUBRAX AV 70 IN) produced by Petróleo Brasileiro S.A. (Petrobrás). This oil has a naphthenic mineral base (molecular formula: C_10_H_8_), inhibited with a BHT antioxidant, for use in transformers of all voltage classes, circuit breakers, and switching equipment. Its production is through a severe hydrotreatment and shows a low power factor associated with high oxidation stability. In the final product, the BHT (DBPC) antioxidant was added in a proportion of 0.3% (w/w). There is no introduction of other additives that are free of contamination with PCBs (polychlorinated biphenyls) or other chlorinated products.

The experiments were performed at room temperature, varying the concentration range from 0 to 9000 ppm of hydrogen gas in the headspace, which was injected into the control chamber for 2 h, followed by 4 h of N_2_ gas injection used as a purge gas to clean the system. These long times were required to promote dissolution and homogeneous distribution into mineral oil. The sensor device was constantly monitored by a Keysight DAQ970A to detect variations in electrical resistance.

Table 3 shows the real concentration of H_2_ dissolved gas in mineral oil, considering the headspace injection parameters for H_2_ gas and its Ostwald coefficient for insulating oil with a naphthenic character, which is 0.0558 at 25 °C and 1 atm.^94^ The Ostwald constant enables the correct quantification of dissolved gases in transformer oil samples; its analysis is complex and critical, and it is determined through chromatography by the ASTM D3612A method.^95^ The Ostwald coefficient is a concept derived from Henry’s law (the concentration of dissolved gas is proportional to the partial pressure in the gas phase) and indicates the solubility of a gas per unit volume at a specific temperature and pressure. The measurement of dissolved gas by measuring the gas in the headspace is accepted as a reliable measurement by the IEC 60567 standard. Several methods of measuring dissolved gas concentration have been developed,^96−99^ but the complexity of these methods does not yet allow for direct measurement, and such methods are mainly used to determine the Ostwald and Henry’s law coefficients.

**Table 3:** Relation Between H_2_ Concentrations Injected into the Headspace (HS) and Dissolved into Mineral Oil (DMO) According to the Ostwald Solubility Coefficient

[H_2_]_HS_ (ppm)	[H_2_]_DMO_ (ppm)	[H_2_]_HS_ (ppm)	[H_2_]_DMO_ (ppm)	[H_2_]_HS_ (ppm)	[H_2_]_DMO_ (ppm)	[H_2_]_HS_ (ppm)	[H_2_]_DMO_ (ppm)
50	2.79	300	16.74	4000	223.2	7000	390.6
100	5.58	400	22.32	5000	279	8000	446.4
200	11.16	500	27.90	6000	334.8	9000	502.2

## Supporting Information Available

Supporting Information Available: The Supporting Information is available free of charge at https://pubs.acs.org/doi/10.1021/acssensors.4c02896.

Detailed scheme of the equipment used for H_2_ dissolution in transformer mineral oil and sensor detection analysis. Photography corresponding to our system for experiments (PDF)
