# Supplementary material for: Nanoengineered PDMS/Pd/ZnO-Based Sensor to Improve Detection of H2 Dissolved Gas in Oil at Room Temperature
Source: ACS Sens. 2025 Apr 4;10(4):2554–68. doi: 10.1021/acssensors.4c02896 (PMC12038843; doi:10.1021/acssensors.4c02896)
Supplement: Supplementary file 1 — se4c02896_si_001.pdf [file se4c02896_si_001.pdf]

SUPPORTING INFORMATION

Nanoengineered PDMS/Pd/ZnO-based sensor to improve detection of H_2 dissolved gas into oil at room temperature

Glauco Meireles Mascarenhas Morandi Lustosa[1,2*], Agnes Nascimento Simões[2], Eugenio de Souza Morita[1,2], André Nunes de Souza[3], Floriano Torres Neto[4], Waldir Antonio Bizzo[2], Talita Mazon[1*].

[1]*Ministério da Ciência, Tecnologia e Inovação (MCTI) – Centro de Tecnologia da Informação Renato Archer, Campinas/SP, 13069-901, Brazil.*

[2]*Universidade Estadual de Campinas (UNICAMP) – Faculdade de Engenharia Mecânica, Campinas/SP, 13083-860, Brazil.*

[3]*Universidade Estadual Paulista (UNESP) – Departamento de Engenharia Elétrica, Bauru/SP, 17033-360, Brazil.*

[4]*HOG, CPFL Geração, Campinas/SP, 13088-900, Brazil.*

**talita.mazon@cti.gov.br*

**glauco.lustosa@cti.gov.br*

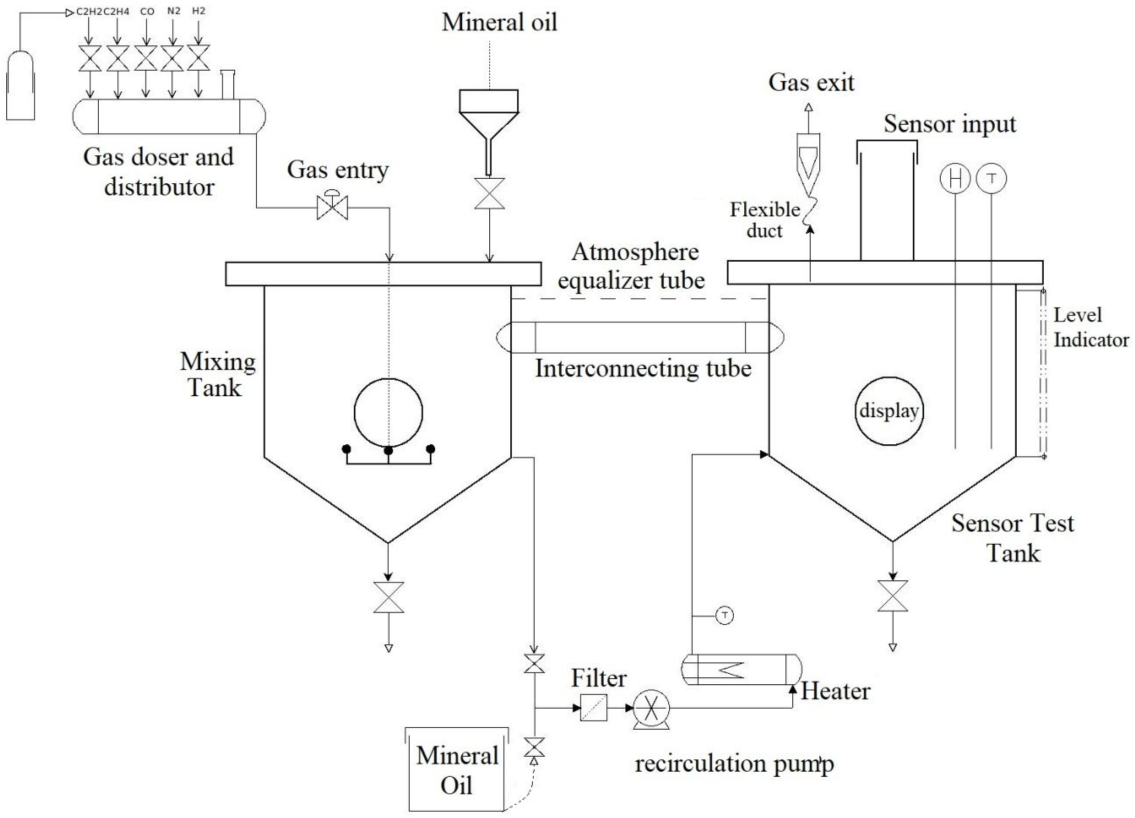

Figure S1. Detailed scheme of the equipment used for H_2 dissolution and analysis in transformer mineral oil.

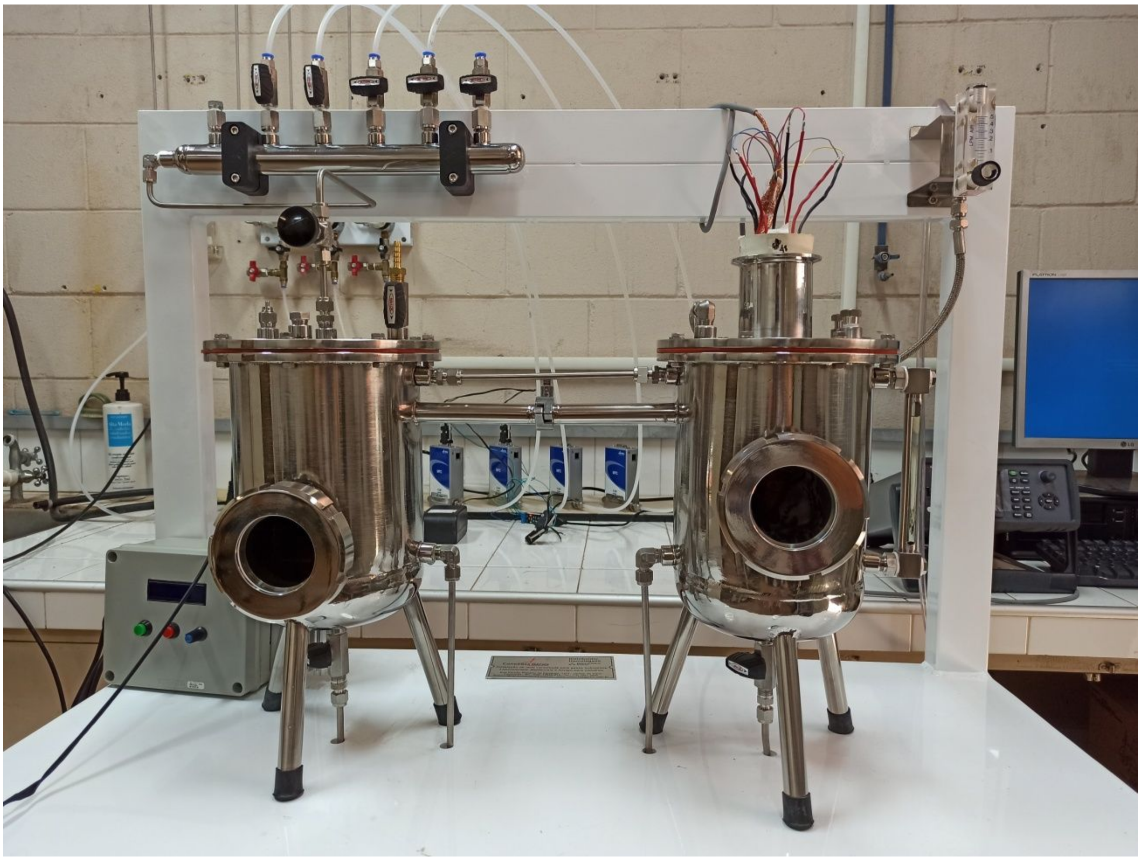

Figure S2. Photo from the Mixing and Sensor Test tanks.
